# Sex Differences in High-Intensity Interval Training–Are HIIT Protocols Interchangeable Between Females and Males?

**DOI:** 10.3389/fphys.2020.00038

**Published:** 2020-01-29

**Authors:** Boris Schmitz, Hannah Niehues, Lothar Thorwesten, Andreas Klose, Michael Krüger, Stefan-Martin Brand

**Affiliations:** ^1^Institute of Sports Medicine, Molecular Genetics of Cardiovascular Disease, University Hospital Muenster, Muenster, Germany; ^2^Department of Physical Education and Sports History, University of Muenster, Muenster, Germany

**Keywords:** high-intensity training, recovery periods, gender, fatigue, repeated-sprint ability (RSA), female

## Abstract

**Background:** High-intensity interval training (HIIT) is a well-established training modality to improve aerobic and anaerobic capacity. However, sex-specific aspects of different HIIT protocols are incompletely understood. This study aimed to compare two HIIT protocols with different recovery periods in moderately trained females and males and to investigate whether sex affects high-intensity running speed and speed decrement.

**Methods:** Fifty moderately trained participants (30 females and 20 males) performed an exercise field test and were randomized by lactate threshold (LT) to one of two time- and workload-matched training groups. Participants performed a 4-week HIIT intervention with two exercise sessions/week: Group 1 (4 × 30,180 HIIT), 30-s all-out runs, 180-s active recovery and Group 2 (4 × 30,30 HIIT), 30-s all-out runs, 30-s active recovery. High-intensity runs were recorded, and speed per running bout, average speed per session, and speed decrement were determined. Blood lactate measurements were performed at baseline and follow-up at rest and immediately post-exercise.

**Results:** Females and males differed in running speed at LT and maximal running speed determined during exercise field test (speed at LT, females: 10.65 ± 0.84 km h^−1^, males: 12.41 ± 0.98 km h^−1^, *p* < 0.0001; maximal speed, females: 14.55 ± 1.05 km h^−1^, males: 17.41 ± 0.68 km h^−1^, *p* < 0.0001). Estimated maximal oxygen uptake was ~52.5 ml kg^−1^ min^−1^ for females and 62.6 ml kg^−1^ min^−1^ for males (*p* < 0.0001). Analysis of HIIT protocols revealed an effect of sex on change in speed decrement (baseline vs. follow-up) in that females showed significant improvements only in the 4 × 30:30 HIIT group (*p* = 0.0038). Moreover, females performing the 4 × 30:30 protocol presented increased speed per bout and average speed per session at follow-up (all *p* ≤ 0.0204), while no effect was detected for females performing the 4 × 30:180 protocol. Peak blood lactate levels increased in all HIIT groups (all *p* < 0.05, baseline vs. follow-up), but males performing the 4 × 30:180 protocol showed no difference in lactate levels.

**Conclusions:** If not matched for physical performance, females, but not males, performing a 4 × 30 HIIT protocol with shorter recovery periods (30 s) present increased average high-intensity running speed and reduced speed decrement compared to longer recovery periods (180 s). We conclude that female- and male-specific HIIT protocols should be established since anthropometric and physiological differences across sexes may affect training performance in real-world settings.

## Introduction

High-intensity interval training (HIIT) has become an increasingly important training modality as it has been shown to improve aerobic and anaerobic capacity with high time efficiency (Sloth et al., 2013; Buchheit and Laursen, 2013a,b; Weston et al., 2014; Milanović et al., 2015). Especially, team sports, such as soccer, basketball, or hockey, are marked by short-duration sprints alternating with (short) recovery periods and performance in team sports thus depends on the ability to perform intermittent exercise and recover from such exercise (Krustrup et al., 2003; Bangsbo et al., 2008). To this respect, improvement of average sprinting performance over a series of sprints and improvement of recovery during relief periods (i.e., reduction of fatigability) is one aim of intermittent sports HIIT protocols (Bishop et al., 2011; Buchheit and Laursen, 2013a).

While a broad range of diverse HIIT protocols exist, HIIT in general can be described as alternating near maximal to supramaximal exercise bouts of short duration ranging from ~10 s to 4 min interspersed with passive or active relief periods of low intensity (Buchheit and Laursen, 2013b; Milanović et al., 2015). Low-volume HIIT with shorter (≤ 30 s) supramaximal or all-out work intervals is often designated as sprint interval training (SIT) (Sloth et al., 2013; Buchheit and Laursen, 2013b). Different parameters including work intensity and duration, relief interval intensity and duration, number of intervals per training session, recovery period between session, and length of the overall HIIT intervention have been discussed as modifiers of HIIT response. In addition, age, training status, and sex have been suggested as additional effectors (Buchheit and Laursen, 2013a,b; Weston et al., 2014; Milanović et al., 2015). Thus, it is still a matter of debate which HIIT protocol may be most effective and how different HIIT protocols affect individual outcome measures. To this respect, it is also essential to investigate the respective response rate to a specific HIIT protocol using an appropriate definition such as the typical error method in addition to reporting significant differences in mean exercise parameters (Alvarez et al., 2017).

Besides the frequent use of HIIT, sex-specific HIIT aspects are incompletely understood and reports on the interchangeability of HIIT protocols and induced training effects between sexes are largely missing from the literature (Gibala et al., 2014; Weston et al., 2014; Hunter, 2016). With respect to repeated high-intensity running, it has been suggested that females, compared to males, exhibit increased fatigue resistance during intermittent sprinting and may present improved recovery despite higher cardiovascular strain and perceived exertion (Laurent et al., 2010, 2014). This observation might in part be based on the finding that sex-dependent differences in intermittent exercise occur during relief periods since females have been reported to present faster ATP recovery (Esbjörnsson-Liljedahl et al., 2002).

Therefore, the purpose of this study was to characterize differences in training performance of two HIIT protocols with different relief periods (30 vs. 180 s) for moderately trained females and males in a real-world setting. We hypothesized that, due to improved fatigue resistance and recovery in females, a HIIT protocol of 4 × 30 s all-out runs with longer recovery periods (180 s) would induce smaller effects on speed decrement in females compared to males.

## Methods

### Study Design

A randomized controlled study design was used to investigate interchangeability of HIIT protocols between unmatched moderately trained females and males (real-world design). Two different time- and workload-matched 4 × 30 s all-out running HIIT protocols were compared: a 4 × 30:180 and a 4 × 30:30 protocols (detailed below). Repeated high-intensity running ability was determined by analysis of 30 s all-out running performance [defined as speed (km h^−1^) per bout] at baseline and in response to the 4-week HIIT intervention. Blood sampling for lactate measurements was performed at rest and immediately post-exercise during baseline (first training session) and follow-up (last training session). Stratified block randomization into the two HIIT groups was performed using sex and individual lactate threshold (LT) determined by a standardized incremental continuous running test (see below). This resulted in an equal number of females in each HIIT group with no differences in physical fitness within each sex. Participants were blinded for the primary outcome measures of the intervention.

### Subjects

Fifty young healthy moderately trained female and male students of the University’s Physical Education Department were recruited at the Institute of Sports Medicine of the University Hospital Muenster. All investigations were performed after the approval of the ethical committee of the medical association Westfalen-Lippe and the Westphalian Wilhelms-University of Muenster (project no. 2013-231-f-S, study acronym SPORTIVA) and in accordance with the declaration of Helsinki. Written informed consent was obtained prior to subjects’ participation in the study. Inclusion criteria were a valid baseline exercise performance test (see below), a health certificate as necessary to study at the University’s Physical Education Department and age > 18 years as reported (Schmitz et al., 2019a). Exclusion criteria were missing adherence to the training program (<6 of 8 training sessions) and injury/illness during the training period or follow-up. Stratified block randomization with sex and performance determined by a standardized maximal performance test as primary parameters was used to allocate participants to one of two training groups. Overall, 12 participants dropped out of the study (females: 3 of the 4 × 30:180 group, 1 of the 4 × 30:30 group; males: 5 of the 4 × 30:180 group, 3 of the 4 × 30:30 group) due to injuries/illness [not associated with the study intervention (*n* = 6)] and missing adherence to the training program (*n* = 6). Previous to the intervention, participants were involved in different types of exercise including team sports, aerobic exercise training, and resistance training. None of the participants had been involved in structured HIIT during 6 months pervious to the intervention, and no participant reported the use of supplementation with known effects on performance. Participants’ diet was not controlled but participants were asked to keep their dietary habits constant, including caffeine and alcohol intake. The use of oral contraceptive hormones in females was asked by questionnaire. Participants were advised to refrain from physical exercise and alcohol intake at least 24 h before each testing or training session.

### Procedures

#### Exercise Parameters

One week before the intervention period, anthropometric data were recorded using standard laboratory equipment. Body composition measurements were performed between 09:00 am 12:00 pm with participants in a well-hydrated state using bioelectrical impedance analysis (MC-780U, Tanita, Arlington Heights, IL, USA). In the second week, all participants performed a standardized incremental continuous running test (ICRT) to determine individual lactate thresholds (LT). The test was performed indoors on a synthetic 200 m running track at ambient temperature (20–22°C, ~60 m above sea level) in groups of four to five participants as described in detail elsewhere with modifications (Léger and Boucher, 1980; Berthoin et al., 1994; Schmitz et al., 2017a,b). In brief, the test started at 8.0 km h^−1^, increasing by 2.0 km h^−1^ every 3 min until total exhaustion of the participant defined as voluntary termination due to fatigue (rating of perceived exertion 19–20 on 6–20 Borg Scale) or failure to reach a track marker twice. The pace was controlled by an automated acoustic device (indicating 25 m track marks), and the test was supervised by at least two experienced trainers and four assistants. To achieve maximal test performance, strong verbal encouragement was provided throughout all tests. Blood was sampled from participants’ earlobes for blood lactate concentration measurements (Biosen S-line, EKF Diagnostics, Magdeburg, Germany) after each interval (3 min). Subjects were fitted with heart rate (HR) monitors combined with a wireless receiver module (Acentas, Muenster, Germany) to determine exercise HR with up to 300 s of recording after the test to assess HR during passive recovery (standing). HR recovery was calculated from delta HR_max_ − HR_3min_ (Schmitz et al., 2019b). Performance at LT (baseline lactate +1.5 mmol L^−1^) was calculated using Winlactat software version 5.0.0.54 (Mesics, Muenster, Germany) as described elsewhere (Roecker et al., 1998; Dickhuth et al., 1999).

#### High-Intensity Interval Training and Determination of Repeated High-Intensity Running Ability

The 4-week training intervention included two matched training groups performing two exercise sessions per week (minimum of 48 h between sessions). All training sessions started with an athletic warm-up for 5 min including light running at 8.0 km h^−1^, knee lifts, heel flicks, and skips. Warm-up was followed by the first 30 s all-out run. A cool-down phase was not included. Training sessions were controlled by at least two experienced trainers and continuously recorded using a digital video camera (Sytuls TG-Tracker, Olympus, Hamburg, Germany).

The training protocols were as follows:

1. Group 1 (4 × 30:180 HIIT): Participants started with an athletic warm-up (5 min) followed by 4 × 30 s sprints (all-out) at maximum speed with 180 s of active recovery periods (light jogging ≤ 8.0 km h^−1^) between bouts (total of three recovery periods).
2. Group 2 (4 × 30:30 HIIT): Participants started with a pre-warm-up (3 × 150 s = 7.5 min of light jogging ≤ 8.0 km h^−1^, time-matched to group 1), followed by an athletic warm-up (5 min) and 4 × 30 s sprints (all-out) at maximum speed with 30 s of active recovery periods between bouts (total of three recovery periods).

The two HIIT protocols were matched for total workload and duration (Table 1; Schmitz et al., 2019c). Respective metabolic equivalents (METs) were estimated according to the Compendium of Physical Activities (Ainsworth et al., 2000) with 8.0 METs for running at 8 km h^−1^ (code 12030, for active recovery) and 19.0 METs for running at all-out speed (code 12132) as reported previously (Schmitz et al., 2018). High-intensity runs were performed on a 200 m indoor running track (track markers placed every 25 m, same facility as ICRT), and maximal performance (m) during sprints was calculated from documented runs (not including the recovery phase). Participants were instructed to run at the highest possible speed they could maintain for each individual 30-s run. In particular, they were instructed not to select an effort they could possibly maintain overall four runs during one session. During all analyzed exercise tests, verbal encouragement was provided to achieve maximal performance. Running speed per bout, average running speed (mean of all four bouts), and speed decrement in percent [over all four bouts, calculated using the equation 100−(mean time/best time × 100), as suggested by Buchheit et al., 2008] were determined. Running speed per bout and average running speed per session were used for within-group comparison. For between-group comparison, speed decrement was used. High-intensity runs were supervised by at least two experienced trainers. Test and retest were scheduled at the identical day of the week and identical daytime with a minimum of 48 h recovery after the last training session. Blood was sampled at baseline and follow-up from participants’ earlobes for blood lactate concentration measurements at rest (before warm-up) and directly after the last run.

**Table 1:** High-intensity interval training (HIIT) workload and recovery by group.

	4 × 30:30 (two sessions/week)				4 × 30:180 (two sessions/week)		
	30-s runs	MET·min·week^−1^	Rec. (s)	Add. Rec. (s)	30-s runs	MET·min·week^−1^	Rec. (s)
Week 1	8	300	180	900	8	300	1,080
Week 2	8	300	180	900	8	300	1,080
Week 3	8	300	180	900	8	300	1,080
Week 4	8	300	180	900	8	300	1,080
Total	32	1,200	4,320		32	1,200	4,320

*MET, metabolic equivalent; Rec., recovery*.

*METs were estimated according to the Compendium of Physical Activities (Ainsworth et al., 2000) with 8.0 METs for running at 8 km h^−1^ (code 12030) and 19.0 METs for running at 19 km h^−1^ (all-out; code 12132). METs for both HIIT groups include warm-up (5 min at ∼8 km h^−1^) and three active recovery phases (180/30 s at ∼8 km h^−1^)*.

### Statistical Data Analysis

Statistical analyses were performed using SPSS v.25 (IBM, Armonk, USA) and GraphPad PRISM V7.0 software (GraphPad Software, La Jolla, USA). Data are presented as mean ± SD or SEM as indicated. Differences between HIIT groups were determined using a sex × group × time repeated-measures ANOVA to identify significant main effects. To address sex differences at baseline, a two-factor (sex and group) repeated-measures ANCOVA with baseline values (speed decrement) as co-variate was used as described (Bagley et al., 2018). Where appropriate, univariate *post hoc* analysis including one-way ANOVA or two-tailed paired *t*-test were performed with Bonferroni’s correction. Linear regression was used to model the relationship between running speed and work/relief intervals of baseline and follow-up training sessions. Data were tested for normal distribution using D’Agostino-Pearson normality test (omnibus K2 test). In case of non-parametric data, Friedman test with Dunn’s *post hoc* correction was used. Data of 38 participants were available (12 females of the 4 × 30,180 group, 14 females of the 4 × 30,30 group, five males of the 4 × 30,180 group, and seven males of the 4 × 30:30 group). Correlation between muscle mass and blood lactate levels were analyzed using Pearson’s product-moment correlation coefficient. Power calculations were performed using G*Power 3.1.9.2 and suggested a necessary sample size of 52 subjects to achieve a statistical power of (1−*β*) = 0.8 at *α* = 0.05 with an estimated effect size of 0.8 for speed decrement as primary outcome parameter. Statistical significance was declared at *p* < 0.05. Responder analysis was performed using the typical error method (TE). TE was calculated for average sprinting speed during a single HIIT session as described using the following equation: TE = SD_diff_/$\sqrt{2}$, where SD_diff_ is calculated as the difference between the variance (SD) of two repeats of the test (Alvarez et al., 2017). Responders were defined as participants who demonstrated an increase greater than 2 × TE away from zero.

## Results

Participants’ anthropometric data and exercise performance parameters at baseline are presented in Table 2. Overall, females and males differed in exercise performance capacity in that speed at LT and maximal running speed determined during baseline ICRT was higher in males (speed at LT, females: 10.65 ± 0.84 km h^−1^, males: 12.41 ± 0.98 km h^−1^, *p* < 0.0001; maximal speed, females: 14.55 ± 1.05 km h^−1^, males: 17.41 ± 0.68 km h^−1^, *p* < 0.0001). Estimation of VO_2max_ from maximal running speed using the equation provided by Léger and Boucher (1980) for the applied field test method suggested ~52.07 ml kg^−1^ min^−1^ and ~52.90 ml kg^−1^ min^−1^ for females and ~61.59 ml kg^−1^ min^−1^ and ~61.62 ml kg^−1^ min^−1^ for males in the 4 × 180:30 and the 4 × 30:30 group, respectively. In addition, males had significantly higher total body muscle mass and leg muscle mass then females (64.64 ± 7.71 kg vs. 45.51 ± 3.03 kg, *p* = 0.0326; 22.16 ± 2.26 kg vs. 14.58 ± 0.88 kg, *p* = 0.0218; Table 2). Males also tended to higher training frequency and duration before the intervention [females: 4.04 ± 1.17 training sessions (289 ± 235 min per week), males: 5.25 ± 1.55 training sessions (420 ± 318 min per week), *p* = 0.0397 for frequency, *p* = 0.106 for duration]. No significant differences in any parameters existed between training groups, neither for females nor for males. Evaluation of questionnaires revealed that the use of oral contraceptive hormones was equally distributed between females of the 4 × 30:30 group and females of the 4 × 180:30 group (71.4 vs. 83.3%).

**Table 2:** Participants’ characteristics at baseline.

	4 × 30:180 HIIT		4 × 30:30 HIIT	
	Females (*n* = 12)	Males (*n* = 5)	Females (*n* = 14)	Males (*n* = 7)
Age, years	23.4 ± 4.4	23.6 ± 1.3	23.1 ± 1.4	22.1 ± 1.8
Height, cm	169.3 ± 5.6	181.2 ± 7.7	171.6 ± 7.56	187.3 ± 7.7
Body mass, kg	61.73 ± 6.71	74.38 ± 8.10	64.86 ± 6.53	83.26 ± 11.07
BMI, kg × m^−2^	21.51 ± 1.94	22.60 ± 0.98	22.09 ± 2.42	23.70 ± 2.56
Muscle mass, kg	44.13 ± 2.59	61.38 ± 6.81	46.89 ± 3.36	67.90 ± 8.25
Leg muscle mass, kg	14.28 ± 0.77	21.06 ± 2.08	14.87 ± 0.97	23.26 ± 2.37
Fat mass, kg	15.26 ± 4.62	9.80 ± 1.58	15.48 ± 3.48	11.84 ± 5.07
Total body water, kg	33.50 ± 1.93	46.50 ± 4.62	35.59 ± 2.52	51.33 ± 5.55
Resting HR, beats·min^−1^	95.5 ± 20.2	95.2 ± 17.7	97.4 ± 19.2	84.0 ± 11.3
Resting LA, mmol·L^−1^	1.3 ± 0.5	1.4 ± 0.4	1.2 ± 0.4	1.2 ± 0.3
Speed at LT, km h^−1^	10.52 ± 0.83	12.62 ± 1.07	10.76 ± 0.87	12.26 ± 0.98
HR at LT, beats·min^−1^	175.7 ± 10.7	171.4 ± 12.4	175.9 ± 13.0	172.6 ± 9.4
Maximal speed, km h^−1^	14.40 ± 0.97	17.40 ± 0.90	14.67 ± 1.14	17.41 ± 0.56
Maximal LA, mmol·L^−1^	12.0 ± 2.1	11.9 ± 1.7	12.1 ± 2.9	11.5 ± 2.4
Maximal HR, beats·min^−1^	195.8 ± 9.9	189.8 ± 8.9	195.4 ± 10.0	192.9 ± 11.4
HR recovery, beats·min^−1^	68.8 ± 12.7	67.4 ± 13.1	63.5 ± 13.8	63.7 ± 8.0

*BMI, body mass index; HR, heart rate; HR recovery, HR_max_ − HR_3min_; LA, blood lactate concentration; LT, individual lactate threshold (baseline lactate +1.5 mmol L^−1^); resting HR, HR determined before start of the exercise test*.

*Data are mean ± SD. Exercise parameters were determined during maximal performance test (incremental running test). Group 4 × 30:180 HIIT, high-intensity interval training with 4 all-out runs of 30-s duration and 180-s active recovery; Group 4 × 30:30 HIIT, high-intensity interval training with 4 all-out runs of 30-s duration and 30-s active recovery. No statistical between-group (i.e., HIIT group) differences were detected*.

### High-Intensity Running Ability

**Figure 1:**
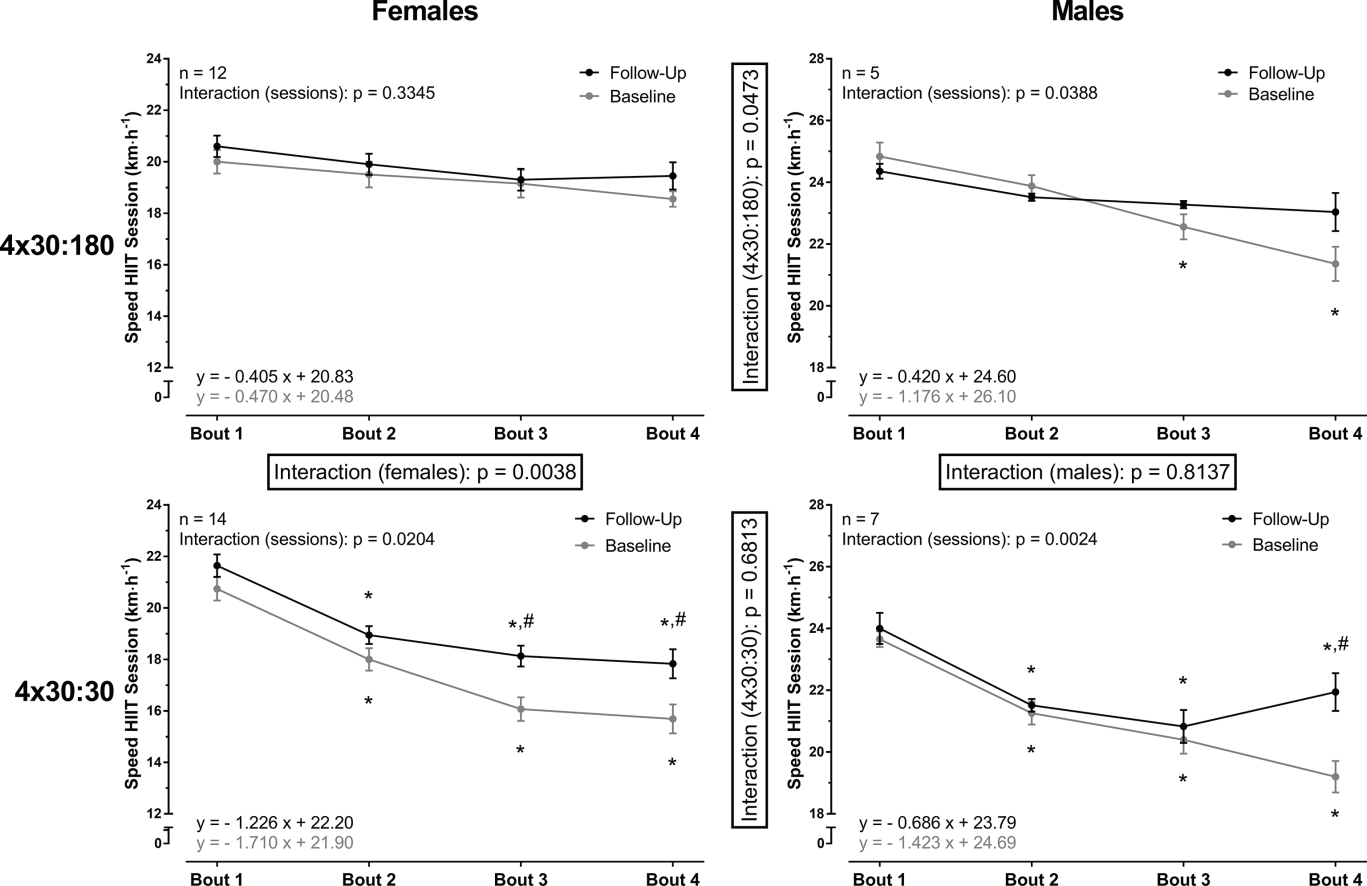
Repeated high-intensity running performance by HIIT protocol and sex. Significant differences were detected for high-intensity running performance between females and males. Females in the 4 × 30:180 group showed no change in speed decrement or running performance at respective bouts. Repeated-measures two-way ANOVA was used to detect interaction effects for change in speed decrement (boxed) and running performance overall bouts. One-way ANOVA was performed where indicated to analyze within-session (^*^p < 0.05, compared to the first running bout) and between-session (^#^*p* < 0.05, comparison of respective bouts at baseline and follow-up) differences. Data are presented as mean ± SEM. Linear regression equations are given in each respective panel.

As shown in Figure 1, there was a significant difference between females and males for change in speed decrement during high-intensity runs. Females performing the 4 × 30:30 protocol showed lowered speed decrement at follow-up (last training session) compared to baseline (first training session; baseline, 15.0 ± 3.6% vs. follow-up, 11.6 ± 3.7%, *p* = 0.004), while no change in speed decrement for females performing the 4 × 30:180 protocol was detected (baseline, 3.7 ± 2.5% vs. follow-up, 4.3 ± 1.4%, *p* = 0.41). For males, no change in speed decrement was observed in any of the two HIIT groups (4 × 30:30 protocol, baseline, 10.7 ± 3.1% vs. follow-up, 7.9 ± 3.8%; 4 × 30:180 protocol, baseline, 6.6 ± 4.7% vs. follow-up, 3.2 ± 2.7%). Since females and males differed in major physical fitness parameters and (leg) muscle mass (Table 2, both *p* < 0.01), which might affect speed decrement during HIIT, speed decrement at baseline was used as co-variate to analyze effects on speed decrement at follow-up, revealing a significant sex × group interaction effect (*p* < 0.001). Responder analysis for females who demonstrated an improvement of speed decrement greater than 2 × TE revealed a response rate of 79% for the 4 × 30:30 protocol. Of note, recorded data also showed that selected speed during the first running bout was not different between baseline and follow-up session in any of the analyzed groups. Comparison of respective running bouts at baseline and follow-up within the 4 × 30:30 group suggested significantly increased speed during the last two high-intensity runs for females at follow-up (bout 3, *p* = 0.0219 and bout 4, *p* = 0.0146), while speed for males was only increased during the last running bout (bout 4, *p* = 0.0008).

### Blood Lactate Concentrations

**Figure 2:**
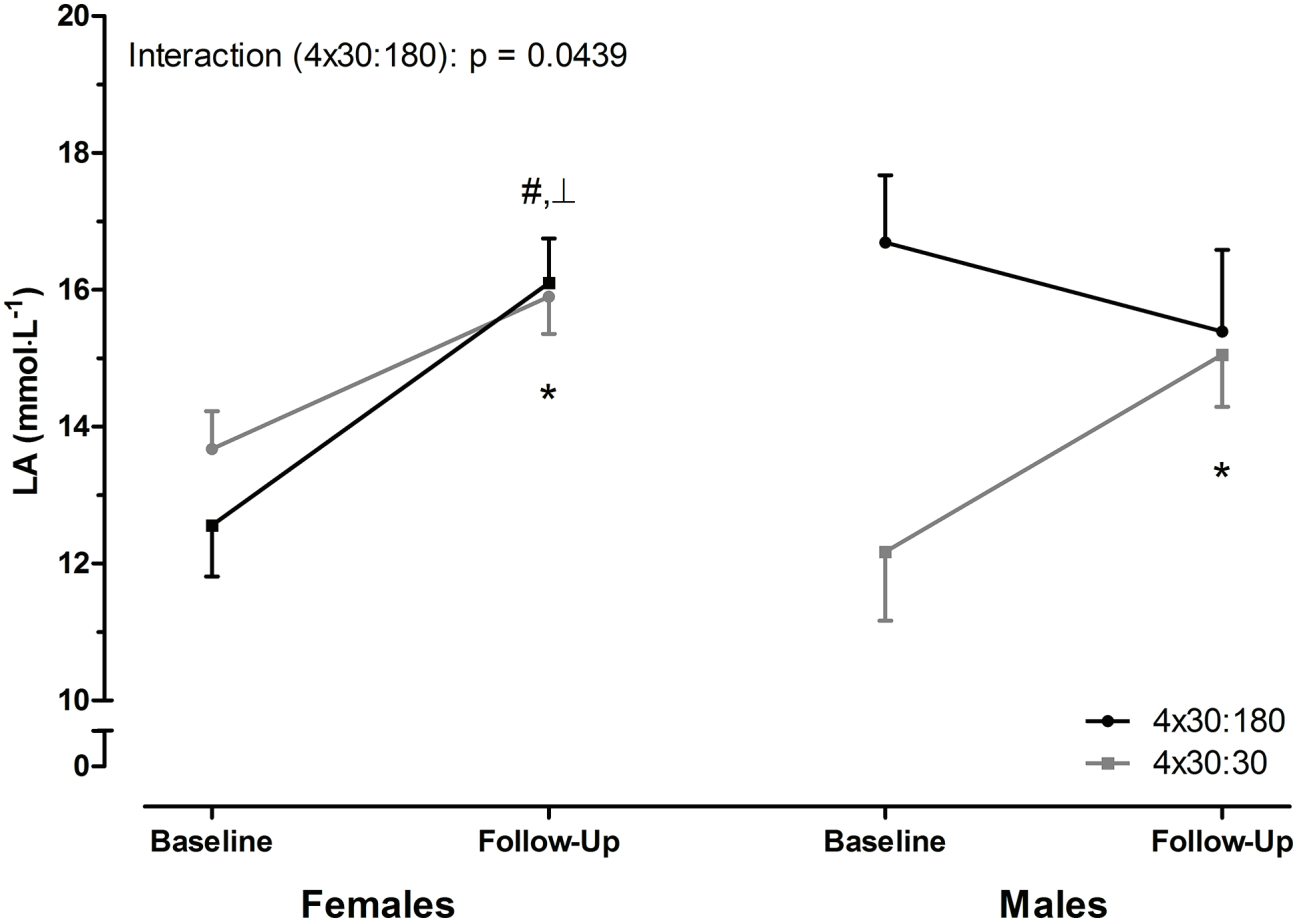
Change in peak blood lactate (LA) concentrations by HIIT protocol. Peak LA concentrations were increased in response to the intervention for females in both groups. In males, peak LA concentrations changed only for the 4 × 30:30 group. Data are presented as mean ± SEM. ^┴^significant interaction by two-way ANOVA for the 4 × 30:180 HIIT protocol. ^#^*p* < 0.05, 4 × 30:180 group, baseline vs. follow-up; ^*^p < 0.05, 4 × 30:30 group, baseline vs. follow-up by paired *t*-test.

As shown in Figure 2, change in peak blood lactate concentrations determined during the first (baseline) and last (follow-up) HIIT session was different between females and males. For the 4 × 30:180 HIIT protocol, a significant sex × time interaction was observed (*p* = 0.0439). While females presented significant changes in peak lactate concentrations in both groups (baseline vs. follow-up, 4 × 30:180 group, *p* = 0.0030; 4 × 30:30 group, *p* = 0.0002), males showed significant change only in the 4 × 30:30 group (*p* = 0.0478). Of note, we observed only a moderate association of leg muscle mass and average speed during HIIT sessions and no association between leg muscle mass and peak blood lactate levels (data not shown).

## Discussion

The current study investigated the repeated high-intensity running ability of moderately trained females and males during two different HIIT protocols. We found that sex significantly affected repeated high-intensity running performance as well as training response. Moreover, HIIT protocol-dependent changes of peak lactate concentrations were different in females and males.

The effects of HIIT on repeated running ability have been investigated in a number of studies including males and females (Edge et al., 2005; Mohr et al., 2007; Cicioni-Kolsky et al., 2013; Purkhús et al., 2016; Viaño-Santasmarinas et al., 2018), but optimal HIIT parameters are still under investigation and sex-specific HIIT protocols are largely missing from the literature. In today’s training practice, HIIT intervention protocols from studies only performed in males are commonly adapted also for females. This practice might, at least to some extent, be erroneous as recent studies have indicated that sex-dependent anthropometric and physiological differences between females and males might significantly affect repeated high-intensity exercise and thus training response (Gibala et al., 2014). In detail, it has been suggested that females may be more resistant to fatigue and have greater ability to recover during repeated bouts of exercise, which represents an important aspect of low-volume interval training. To this respect, it is of interest that sex has been documented to affect both, central and peripheral mechanisms of skeletal muscle fatigue (see Billaut and Bishop, 2009 for comprehensive review). With respect to central fatigue (i.e., affecting mechanisms at the central nervous system), it has been reported that during maximal intermittent isometric contractions greater reduction in voluntary muscle activation can be observed in males compared to females (Russ and Kent-Braun, 2003) and a decline in skeletal muscle recruitment after heavy-resistance exercise in males combined with slower acute recovery compared to females has been detected using electromyography (Häkkinen, 1993). In terms of sex-dependent peripheral fatigue, it has been reported that greater peripheral fatigue in males vs. females may occur during sustained (120 s) isometric maximal voluntary contraction of the knee extensor muscles (Solianik et al., 2017) and maximal voluntary knee extensor torque decreased more in males than in females after a 110-km ultra-marathon (Temesi et al., 2015). Of note, the latter two studies did not report any sex-dependent differences on central fatigue (Temesi et al., 2015; Solianik et al., 2017), indicating that sex-related differences in fatigue may occur without effects on central fatigue. Based on these reports, the current study investigated if HIIT recovery periods and thus altered work/rest ratio would lead to sex-dependent differences in speed decrement. Our data indicate that females performing a 4 × 30:180 all-out running HIIT showed a higher level of recovery during the relief period compared to males. Of note, this improved recovery seemed to reduce the adaptive training response of females performing this protocol and only the 4 × 30:30 protocol lead to significant changes in speed decrement and increased speed per running.

With respect to repeated high-intensity running performance, our findings are in line with Laurent et al. (2010), who reported that females also exhibited significantly lower performance decrement and thus increased fatigue resistance during intermittent sprinting (three bouts of eight all-out 30-m sprints, 5 min of rest). The same group later used a setting of self-paced HIIT (HR_max_ 92–97%, RPE 13–14; three bouts of six 4-min runs with 1, 2, or 4 min recovery with 15–20 min of rest between bouts) and reported that females presented improved recovery despite higher cardiovascular strain and perceived exertion (Laurent et al., 2014). The observed sex-dependent differences might be explained by differences in the aerobic vs. anaerobic contribution to the work performed during high-intensity exercise based on overall sex-specific muscle characteristics (Billaut and Bishop, 2009). Esbjörnsson-Liljedahl et al. (2002) investigated the acute metabolic response to repeated sprint exercise (three 30-s cycle sprints, 20 min of rest between sprints) in females and males and reported that in type I muscle fibers, glycogen reduction was smaller in females compared to males and was accompanied by lower accumulation of plasma ATP breakdown products. In type II muscle fibers, smaller reduction of ATP and reduced accumulation of inosine monophosphate was observed in females. Of note, the group reported no sex differences in ATP changes and accumulation of breakdown products during the exercise bouts and concluded that sex-dependent differences occur during recovery periods of intermittent exercise and that females might possess a faster recovery of ATP (Esbjörnsson-Liljedahl et al., 2002). This observation is partly consistent with our observation that at baseline, peak blood lactate levels in females performing the 4 × 30:180 protocol were lower compared to males (despite comparable levels during baseline ICRT) and average sprinting speed remained unchanged in females performing the 4 × 30:180 protocol, indicating better recovery and lower fatigability.

Since greater initial strength (as usually observed in males) can be associated with increased fatigability for some muscle groups and specific tasks (Hunter, 2014), it has been suggested that sex-dependent differences in terms of percent work decrement and electromyography change can be attenuated when females and males are matched for initial-sprint work and it has been concluded that that greater fatigability during intermittent sprinting in men may be a consequence of greater absolute initial work (sprinting speed) (Billaut and Bishop, 2012). While this might explain the within HIIT group differences between females and males observed in our series, between group differences (i.e., work/relief-dependent differences) observed for females without differences in initial sprinting speed are likely based on different mechanisms. Moreover, additional analyses including speed decrement values at baseline as co-variate also suggested a significant group × sex interaction effect in our series. In addition, there is evidence that sex-specific differences in fatigue and ability to recover during sprinting exist even if matching for exercise parameters such as VO_2max_ is performed (Astorino et al., 2011; Mageean et al., 2011). It may be concluded that since recovery during repeated sprints depends on aerobic processes, improved recovery in females may be associated with a greater aerobic contribution to recovery. To this respect, there is evidence that females have greater muscle perfusion than men (also during exercise at identical relative intensity), leading to improved oxygen delivery to the muscle and improved removal of metabolites that interfere with the muscle contractile function and limit voluntary activation by increased peripheral afferent feedback (Hunter, 2014).

HIIT in general is able to improve lactate anion and H^+^ removal from the working muscle and increase muscle fiber levels of the monocarboxylate symporter MCT-1 (*SLC16A1*) and the Na^+^/H^+^ exchanger NHE-1 (*SLC9A1*) in males and females (Juel et al., 2004; McGinley and Bishop, 2017). Interestingly, recent data suggested that NHE-1 levels might be affected by rest interval durations (McGinley and Bishop, 2017). In the current study, we determined lactate concentrations in the blood, which are affected by rates of production, removal, and uptake and do not allow direct conclusions on muscle lactate levels (Buchheit and Laursen, 2013a). However, blood lactate levels at baseline and follow-up were not affected by work/relief ratio in females despite longer recovery duration in the 4 × 30:180 group, which, at the observed higher running speeds during the 4 × 30:180 program and identical removal and uptake rates, would suggest uptake and removal of higher amounts of lactate from the blood and the muscle, respectively. This is of interest as muscular lactate anion production during exercise is accompanied with increased H^+^ accumulation and thus muscular fatigue (Juel et al., 2004; McGinley and Bishop, 2017). Thus, it is conceivable that moderately trained females performing a 4 × 30:180 all-out running HIIT show a higher level of recovery during the relief period compared to males. This seems to reduce the adaptive training response and might therefore be suboptimal for improving speed decrement and running speed per bout at least when compared to a 4 × 30:30 HIIT protocol.

### Limitations

It has been shown that HIIT effects on aerobic exercise capacity in terms of change in VO_2max_ may not be different when males and females were matched for VO_2max_ at baseline (Astorino et al., 2011). We did not match female and male participants for any physical fitness parameter since the current study aimed to investigate if different HIIT protocols are interchangeable between females and males in a “real-world design.” It may thus be argued that the here described effects do not depend on the biology of sex but are rather influenced by physiological differences. We also did not control the menstrual cycle of our female participants or oral contraceptive cycle phase and estrogen levels. This might be of importance as some studies on repeated short-term high-effort/all-out performance have suggested improved recovery during exercise and enhanced blood lactate removal from the working muscle as well as increased O_2_ uptake during recovery during the luteal phase (Middleton and Wenger, 2006). However, the effects of the different phases of the menstrual cycle or oral contraceptive cycle phase on intermittent-exercise/repeated sprinting and performance and muscle fatigue in general is still a matter of debate and requires further investigations (Billaut and Bishop, 2009; Rechichi et al., 2009). The number of males in our study involved in the final analyzes did not equal the number of analyzed females, which lead to a reduction in statistical power to detect significant differences. Our results will thus have to be confirmed in a study with equal samples sizes for females and males. Furthermore, our study did not involve an independent test for change in repeated high-intensity exercise performance outside of the reported training data. Thus, we cannot conclude that intermittent exercise capacity in general has been improved.

## Conclusions

We conclude that HIIT protocols may not be used interchangeably between females and males without restriction when differences in exercise capacity are present. Female-specific HIIT protocols aiming at improvement of repeated running ability may need to consider reduced fatigability and improved recovery of females and should include shorter recovery periods. This might be of importance predominantly in intermittent sports such as soccer and basketball in which high-intensity actions and recovery determine competitive game performance.

## Data Availability Statement

All datasets generated for this study are included in the article/supplementary material.

## Ethics Statement

The studies involving human participants were reviewed and approved by the ethical committee of the medical association Westfalen-Lippe and the Westphalian Wilhelms-University of Muenster. The patients/participants provided their written informed consent to participate in this study.

## Author Contributions

BS designed and supervised the study. BS, AK, and HN enrolled participants, performed exercise testing, and supervised the training interventions. BS and HN analyzed training and testing data, interpreted results, and wrote the manuscript. LT, MK, and S-MB reviewed the manuscript and provided important intellectual content. All authors approved the final version of the manuscript.

### Conflict of Interest

The authors declare that the research was conducted in the absence of any commercial or financial relationships that could be construed as a potential conflict of interest.
